# UDP-Glucuronosyltransferase 1A Determinates Intracellular Accumulation and Anti-Cancer Effect of β-Lapachone in Human Colon Cancer Cells

**DOI:** 10.1371/journal.pone.0117051

**Published:** 2015-02-18

**Authors:** Huiying Liu, Qingran Li, Xuefang Cheng, Hong Wang, Guangji Wang, Haiping Hao

**Affiliations:** State Key Laboratory of Natural Medicines, Key Lab of Drug Metabolism and Pharmacokinetics, China Pharmaceutical University, Nanjing, China; Boston University Goldman School of Dental Medicine, UNITED STATES

## Abstract

β-lapachone (β-lap), an NAD(P)H:quinone oxidoreductase 1 (NQO1) targeting antitumor drug candidate in phase II clinical trials, is metabolically eliminated via NQO1 mediated quinone reduction and subsequent UDP-glucuronosyltransferases (UGTs) catalyzed glucuronidation. This study intends to explore the inner link between the cellular glucuronidation and pharmacokinetics of β-lap and its apoptotic effect in human colon cancer cells. HT29 cells S9 fractions exhibited high glucuronidation activity towards β-lap, which can be inhibited by UGT1A9 competitive inhibitor propofol. UGT1A siRNA treated HT29 cells S9 fractions displayed an apparent low glucuronidation activity. Intracellular accumulation of β-lap in HCT116 cells was much higher than that in HT29 cells, correlated with the absence of UGT1A in HCT116 cells. The cytotoxic and apoptotic effect of β-lap in HT29 cells were much lower than that in HCT116 cells; moreover, β-lap triggered activation of SIRT1-FOXO1 apoptotic pathway was observed in HCT116 cells but not in HT29 cells. Pretreatment of HT29 cells with UGT1A siRNA or propofol significantly decreased β-lap’s cytotoxic and apoptotic effects, due to the repression of glucuronidation and the resultant intracellular accumulation. In conclusion, UGT1A is an important determinant, via switching NQO1-triggered redox cycle to metabolic elimination, in the intracellular accumulation of β-lap and thereafter its cytotoxicity in human colon cancer cells. Together with our previous works, we propose that UGTs determined cellular pharmacokinetics is an important determinant in the apoptotic effects of NQO1 targeting substrates serving as chemotherapeutic drugs.

## Introduction

UDP-glucuronosyltransferases (UGTs) are major phase II drug metabolizing enzymes that catalyze the glucuronidation of numerous endogenous compounds such as bilirubin, bile acids, thyroid hormone, and steroid hormones as well as substantial exogenous substrates including therapeutic drugs, carcinogens, and environmental pollutants. UGTs are considered as an important detoxification system for its ability to promote the metabolic elimination and thus the biological efficacies of the substrates [1–3]. Moreover, the polymorphisms in the UGT genes are closely associated with the risk and incidence of various types of diseases [4]. Although the role of UGTs in chemotherapeutic resistance has been commonly acknowledged, the direct link between the intracellular drug accumulation with chemotherapeutic efficacy remains to be established [5,6]. More recently, we have clarified that an NQO1 substrate, Tanshinone IIA, presents a two-electron reduction by NQO1 producing a highly reactive catechol metabolite that can be quickly glucuronidated with UGTs presence. However, if UGTs are absent or their glucuronidation activities are inhibited, the highly unstable catechol intermediate may turn back to Tanshinone IIA automatically, thus forming a redox cycle of quinone reduction and auto-oxidation, in which a large amount of reaction oxygen species (ROS) are produced [7]. These results suggested that UGTs may act in switching NQO1 triggered apoptotic effects to metabolic elimination in cancer cells and thus may be involved in the intrinsic chemoresistance of NQO1 targeting anti-cancer agents. To further validate this important concept, we extended our study to another NQO1 targeting agent.

β-lapachone (β-lap; 3,4-dihydro-2,2-dimethyl-2H-naphtol[1,2-b]pyran-5,6-dione) is a naturally occurring naphthoquinone from lapacho tree (Tabebuia avellanedae), known to have various pharmacological activities including antiviral, antiprotozoal and anticancer effects [8–11]. It has been demonstrated that β-lap exhibits strong cytotoxicity toward a variety of animal and human cancer cell lines [12–14], and can synergistically kill cancer cells when combined with paclitaxel (Taxol), ionizing radiation and heat shock [15–17]. Numerous hypotheses of the mechanism of β-lap–induced cell death were proposed and amounts of evidence promoted β-lap as a promising candidate for cancer therapy [10,18–21].

Since β-lap is also predominantly eliminated via NAD(P)H: quinone oxidoreductase 1 (NQO1) and subsequent UGT catalyzed metabolism [7,22], we assumed that the expression and activity of UGTs in cancer cells could be also an important determinant in the anti-cancer effect of β-lap, similar to that observed from our previous study of Tanshinone IIA. In view that β-lapachone is currently on phase II clinical trials, the validation of whether UGTs mediated cellular metabolism could induce intrinsic resistance is very important to its further development and its future clinical use as a chemotherapy drug. β-lap-induced cell death was characterized by dramatic ROS formation, cell cycle arrest in G_0_/G_1_, extensive DNA damage, depletion of NAD^+^/ATP levels, hyperactivation of poly(ADP-ribose)polymerase-1 (PARP-1) and proteolytic cleavage of PARP-1 [10]. The present study focuses on elucidating the role of UGTs in determining intracellular accumulation, ROS formation, apoptotic effect and SIRT1-FOXO1 pathway activation of β-lap in human colon cancer cells.

## Experimental Section

### Cell Lines and Transfections

Human colon cancer cell lines HT29 and HCT116 were obtained from the American Type Culture Collection (ATCC, USA). Cells were grown in McCoy’s 5a with 10% FBS, 100 U/mL penicillin, and 100 mg/mL streptomycin at 37°C in a humidified incubator with 5% CO_2_.

Cells were transfected with UGT1A siRNA or negative control siRNA (Invitrogen) using lipofectamine RNAiMAX transfection reagent (Invitrogen) according to the manufacturer's reverse transcription protocol.

### Chemicals and Reagents

β-lapachone was obtained from Southeast Pharmaceuticals, Inc. (Jiangsu, China). Propofol, N-acetyl cysteine (NAC), dicoumarol (DIC), 3-(4,5-dimethylthiazol-2-yl)-2,5-diphenyltetrazolium bromide (MTT), glucose 6-phosphate, glucose 6-phosphate dehydrogenase, β-nicotinamide adenine dinucleotide phosphate (NADP), uridine 5’-diphosphate-glucuronic acid (UDPGA), D-saccharic acid 1,4-lactone, β-D-glucuronidase and chlorzoxazone, were all purchased from Sigma Aldrich. Diazepam was obtained from the National Institute for the Control of Pharmaceutical and Biological Products (Beijing, China).

### Quantitative RT-PCR Assay

Total mRNA was isolated using TRIzol (Invitrogen) and reverse transcribed to cDNA according to the manufacturer’s protocol (Takara). qRT-PCR was performed with SYBR Premix ExTaq II (Takara) in a reaction volume of 10 μL. PCR conditions were 95°C for 1 min, 40 cycles of 95°C for 5 seconds, 60°C for 30 seconds, and 72°C for 30 seconds. β-Actin gene was used as an endogenous control.

### Western Blot Assay

For Western blotting, cells were extracted in lysis buffer, resolved by SDS-PAGE and transferred to nitrocellulose membranes. Proteins were detected using specific primary antibodies directed against UGT1A (H-300, 1:200, Santa Cruz Biotechnology, USA), SIRT1 (H-300, 1:200, Santa Cruz Biotechnology, USA), Ac-FOXO1 (D-19, 1:200, Santa Cruz Biotechnology, USA), FOXO1 (C29H4, 1:1000, Cell Signaling, USA), TRAIL (C92B9, 1:1000, Cell Signal Technology, USA), Bim (C34C5, 1:1000, Cell Signal Technology, USA), FasL (1:200, BD pharmingen, USA) or BCL-6 (N-3, 1:200, Santa Cruz Biotechnology, USA). The protein expression levels were normalized with GAPDH (1:5000, shengxing, China). After washing with TBST, the membrane was incubated with HRP-conjugated secondary antibody (1:10000, KeyGen, China) for 1 h. The signal was detected by enhanced cheniluminescence (ECL, Millipore).

### Glucuronidation Assay of β-lap in S9 Fractions

The enzyme kinetics assay for β-lap (0.0625−3 μM) glucuronidation was conducted in HT29 S9 (0.005 mg). HT29 cells were harvested, resuspended with phosphate buffered saline, sonicated and then centrifugated at 9000 g for 20 min. Supernatant was collected as S9 fractions. For propofol inhibition study, propofol (0–400 μM) was co-incubated with β-lap (0.5 μM) at 37°C for 10 min. The enzyme kinetic analysis were performed as described above with the method based on our previous report [22].

### Intracellular Accumulation and Glucuronidation of β-lap in Viable Cells

Cells with 80% confluence were exposed to 5 μM β-lap for 10, 20, 30, 60, 90, 120 min. Both cells and culture medium were collected at indicated time. Cells were harvested and washed for three times by ice-cold physiological saline. 300 μL of ultrapure water was added to each well and freezing/thawing for three times. Each sample was added with three fold of ice-cold acetonitrile, vortexed for 5 min and centrifugated at 18000 rpm for 10 min. Supernatant was obtained and analyzed by LC-MS method based on our previous report [22].

### ROS Assay and GSSG-GSH Ratio Assay

Cells were incubated with 10 μM/L of DCFH-DA in the loading medium at 37°C for 30 min. After DCFH-DA was removed, cells were collected and 500 μL of lysis buffer (0.1N NaOH in 50% MeOH) was added. Spin at 6000 rpm for 1 min. Transfer 200 μL of cell lysate to measure the fluorescence intensity using fluorescence plate reader. The excitation filter was set at 488 nm and the emission filter was 525 nm. Total glutathione and GSSG were measured according to GSH and GSSG Assay Kit (Beyotime, Jiangsu, China). The absorbance changes at 412 nm were detected with spectrophotometer. The amount of GSSG and GSH was obtained separately according to standard curve and their ratio was then calculated.

### Cytotoxicity Assay

Cells with 80% confluence were exposed to 0 μM, 1.25 μM, 2.5 μM, 5 μM, 10 μM or 20 μM of β-lap. After cultivated for 24 h, 20 μL of MTT was added to each well and incubated for 4 h at 37°C. MTT solution was removed and 150 μL DMSO was then added to dissolve MTT crystals. Absorbance at 570 nm was obtained with spectrophotometer after agitating the plates for 10 min on a shaker.

### Apoptosis Assay

Cells with 80% confluence were exposed to 5 μM of β-lap for 24 h and washed by ice-cold PBS. Apoptosis was quantified by using APO-BRDU Kit (BD Biosciences, USA). Cells were stained according to the manufacturer’s instruction. Labeled cells were analyzed with flow cytometry FACS Calibur (BD Biosciences, USA).

### Statistical analysis

Data are presented as mean ± SEM. Student’s t-test or Mann–Whitney U tests were performed using Prism software. Statistical signiﬁcance in t-test are plotted as following: *P<0.05, **P<0.01 and ***P<0.001.

## Results

### UGT1A Determines β-lap Glucuronidation in HT29 Cell S9 Fractions

According to our previous study [22], the quinone reduction and subsequent monoglucuronidation to produce a pair of regioisomers (M1 and M2) is the predominant metabolic pathway of β-lap. Moreover, UGT1A9 and UGT2B7 are the dominant isozymes responsible for β-lap glucuronidation [22]. We thus first evaluated the UGTs expression levels in both HT29 and HCT116 cell lines. Real-time PCR assays showed that multiple UGT1A genes (UGT1A3, UGT1A4, UGT1A6, UGT1A7 and UGT1A9) are positively expressed in HT29 cells but not in HCT116 cells, consistent with previous reports [7]. However, UGT2B7, which is dominantly responsible for the formation of β-lap glucuronidation metabolite M1 [22], was undetectable in both HT29 and HCT116 cells. A high expression level of UGT1A in HT29 cells and the absence of UGT1A in HCT116 cells were supported by western blot assay. UGT1A siRNA sharply decreased the mRNA levels of multiple UGT1A and the total UGT1A protein expression in HT29 cells.

**Figure 1:**
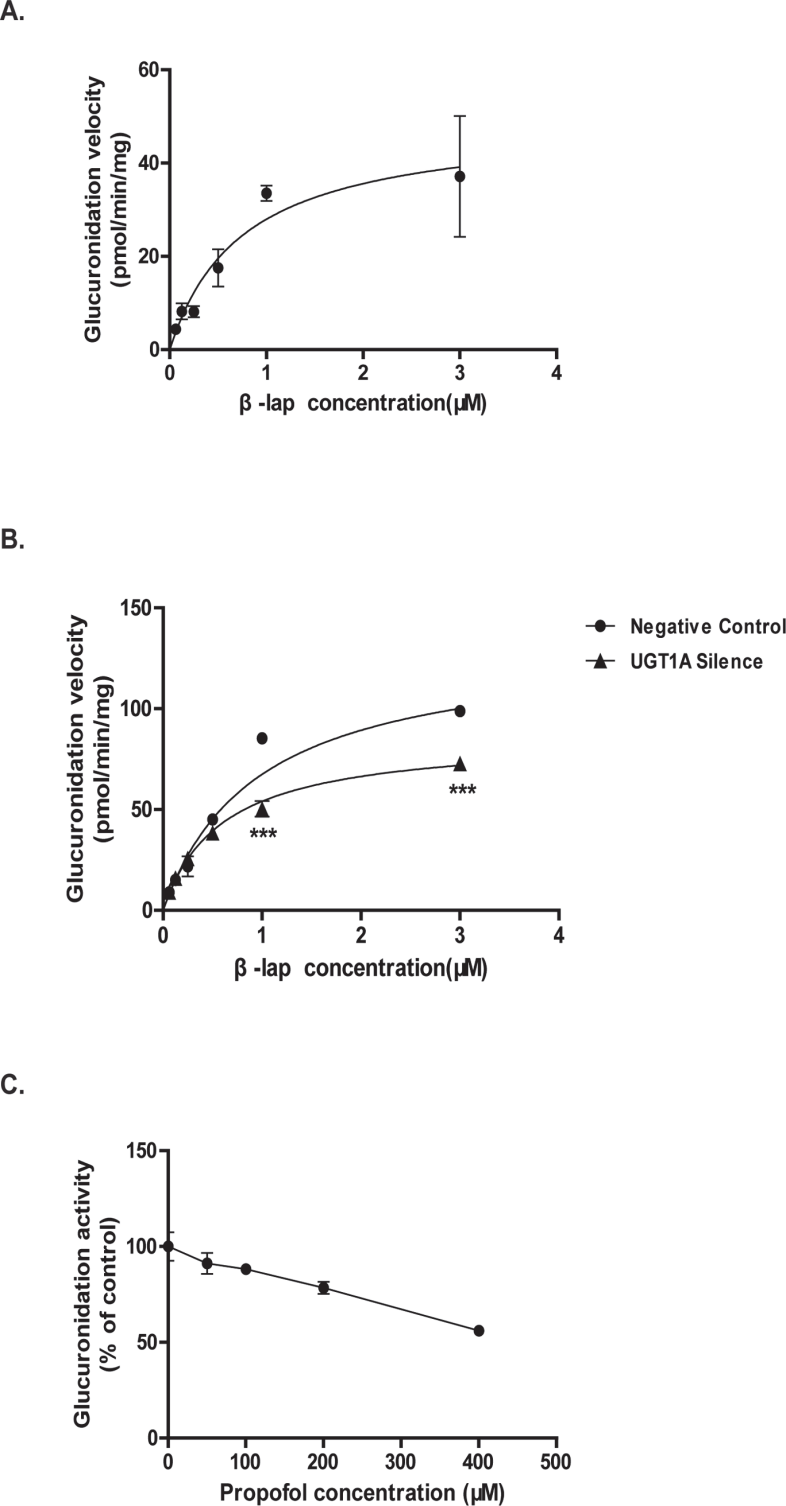
Glucuronidation of β-lap in HT29 cell S9 fractions. HT29 cells S9 were incubated with β-lap according to the details in methods. (A) A typical Michaelis-Menten kinetics of β-lap glucuronidation in HT29 cells S9 fractions; (B) UGT1A siRNA treated HT29 cells S9 fractions significantly decreases β-lap glucuronidation; (C) Inhibitory potency of propofol on β-lap glucuronidation in HT29 cell S9 fractions. Results are presented as mean ± 3 SEM of three independent experiments (***P<0.001, UGT1A siRNA treatment vs. negative control cells).

Consistent with the UGTs expression profile, M2 was detected in HT29 cell S9 fractions while M1 was undetectable, which can be explained with the absence of UGT2B7 in the colon cancer cells. Enzyme kinetic assay shows that β-lap glucuronidation exhibited a typical Michaelis-Menten kinetics in S9 fractions prepared from HT29 cells (Fig. 1A). UGT1A silencing led to β-lap glucuronidation inhibition manifested with a significant decrease of maximum velocity (V_max_) and intrinsic clearance (CL_int_, V_max_/K_m_) values but little influence in the apparent K_m_ values for the production of M2 (Fig. 1B, Table 1). As a UGT1A9 specific substrate [23], propofol showed dose-dependent inhibition on the production of M2 in HT29 cells (Fig. 1C).

**Table 1:** Best-fit enzyme kinetics parameters for β-lap glucuronide (M2) formation in siRNA treated HT29 cell S9 fractions.

	Km (μM)	Vmax (pmol/min/mg protein)	CLint (μl/min/mg protein)
Negative Control	0.78±0.01	130.09±2.23	166.45±0.42
UGT1A Silence	0.63±0.06	86.61±2.18***	138.83±9.18*

Data are shown as mean ± SEM of three independent experiments; *P<0.05, ***P<0.001, UGT1A Silence vs. Negative Control.

### UGT1A Compromises β-lap Accumulation in Colon Cancer Cells

**Figure 2:**
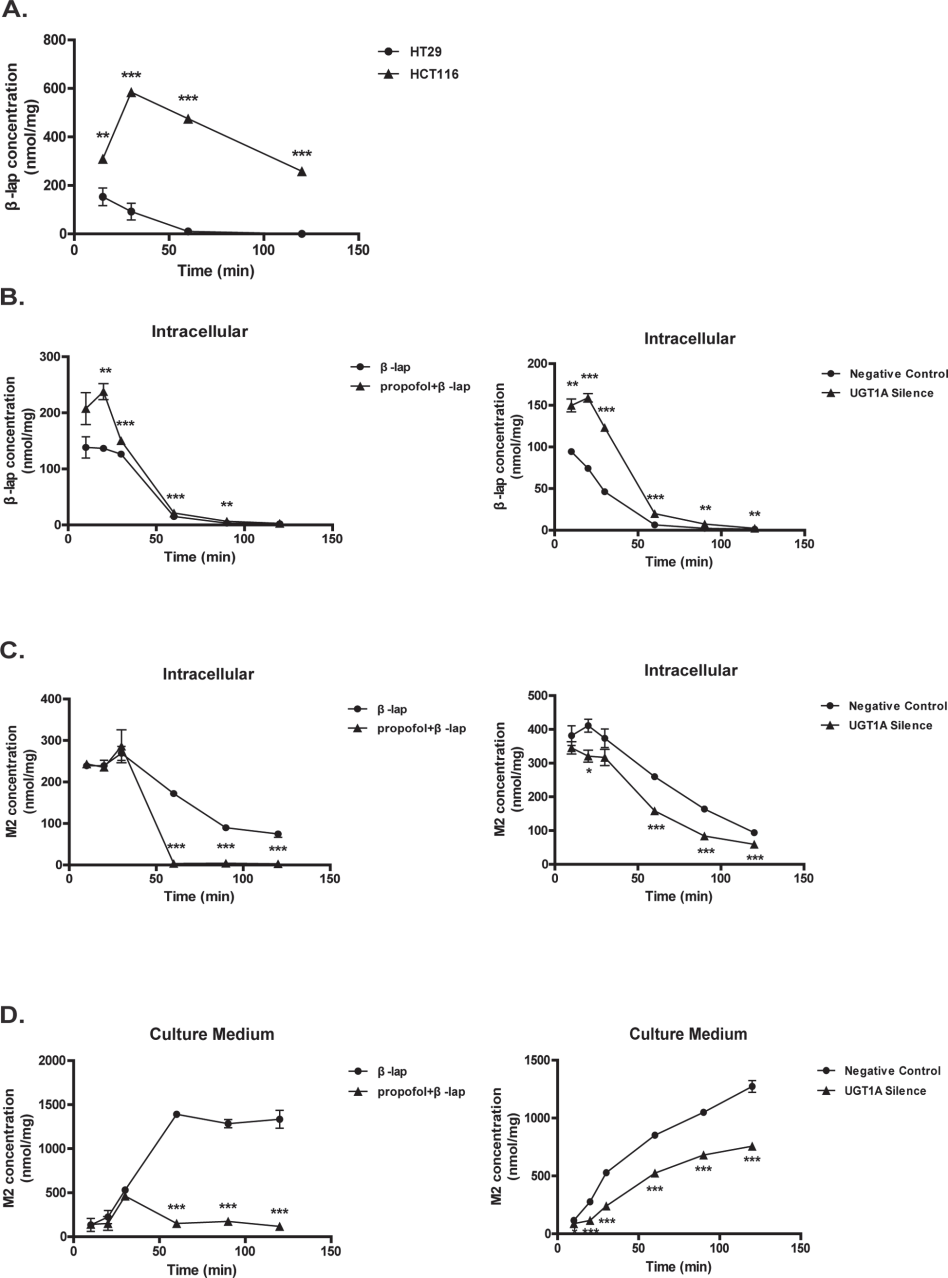
Intracellular accumulation and glucuronidation of β-lap in colon cancer cells. HT29 cells were pretreated with propofol (100 μM) for 1 h or UGT1A siRNA for 24 h. Cells were exposed to β-lap (5 μM) for indicated time. (A) Intracellular β-lap level in HCT116 is much higher than in HT29 cells; (B) Propofol or siRNA pretreatment lead to intracellular accumulation of β-lap in HT29 cells; (C) Propofol or siRNA pretreatment decreases intracellular M2 level in HT29 cells; (D) Propofol or siRNA pretreatment decreases M2 level in HT29 cell culture medium. Data are shown as mean 3 ± SEM of at least three independent experiments (*P<0.05, **P<0.01, ***P<0.001, propofol or UGT1A siRNA pretreatment vs. control cells).

A cellular pharmacokinetic study was performed to test whether UGT1A can influence β-lap accumulation in the living cells. The dynamic intracellular level of β-lap and its metabolite M2 in HT29 and HCT116 cells were examined at different time point of β-lap exposure. Of interest, β-lap gained a very high level at 20 min and then decreased slowly in HCT116 cells. In contrast, the elimination of β-lap in HT29 cells was much faster (Fig. 2A). Both the area under curve from 0 to 120 min (AUC_0–120 min_) and maximum concentration (C_max_) of β-lap in HT29 were significantly lower than in HCT116 cells (Table 2). Pretreatment with propofol or UGT1A siRNA in HT29 cells led to a significant higher level of β-lap, evidenced by both AUC_0–120 min_ and C_max_ values (Fig. 2B, Table 2, Table 3). Consistently, M2 was detectable in HT29 ever since β-lap treatment, indicating a fast glucuronidation of β-lap. Intracellular M2 level peaked at 20 min and then dropped (Fig. 2C), while M2 level in culture medium increased continuously over the entire duration (Fig. 2D). Pretreatment with either propofol or UGT1A siRNA transfection reduced M2 formation in HT29 cells as well as in the culture medium (Fig. 2C and 2D, Table 2, Table 3).

**Table 2:** AUC_0–120 min_ and C_max_ values of β-lap and its glucuronide (M2) with or without propofol pretreatment in HT29 and HCT116 cells and culture medium.

		AUC_0–120 min_(min nmol/mg protein)		C_max_(nmol/mg protein)	
		β-lap only	β-lap+propofol (100 μM)	β-lap only	β-lap+propofol (100 μM)
In cells	β-lap(HT29)	6136.84±230.51	8769.42±745.76**	165.15±19.46	259.27±26.93**
	M2(HT29)	18352.94±1727.61	10786.04±1344.39**	269.01±29.20	299.61±45.46
	β-lap(HCT116)	46909.92±581.29^###^	-	584.60±15.06^###^	-
In medium	M2(HT29)	114472.52±3974.31	21732.26±3325.10***	1427.50±41.81	414.92±114.75***

Data are shown as mean ± SD of three independent experiments; **P<0.01, ***P<0.001, propofol pretreatment vs.β-lap only; ###P<0.001, HCT116 vs. HT29.

**Table 3:** AUC_0–120 min_ and C_max_ values of β-lap and its glucuronide (M2) with or without UGT1A siRNA pretreatment in HT29 and HCT116 cells and culture medium.

		AUC_0–120_(min nmol/mg protein)		C_max_(nmol/mg protein)	
		β-lap+Scrambled siRNA	β-lap+UGT1A siRNA	β-lap+Scrambled siRNA	β-lap+UGT1A siRNA
In cells	β-lap(HT29)	2891.48±181.33	6405.59±335.70***	94.41±4.20	159.00±8.80***
	M2(HT29)	29517.53±912.22	21157.63±891.29**	411.17±19.14	345.15±18.29
In medium	M2(HT29)	104405.40±4633.90	54345.81±2015.78***	1190.00±56.74	756.46±31.24***

Data are shown as mean ± SEM of three independent experiments; **p<0.01, ***p<0.001, UGT1A silence group vs. negative control group.

### UGT1A Eliminates β-lap -induced ROS Formation

**Figure 3:**
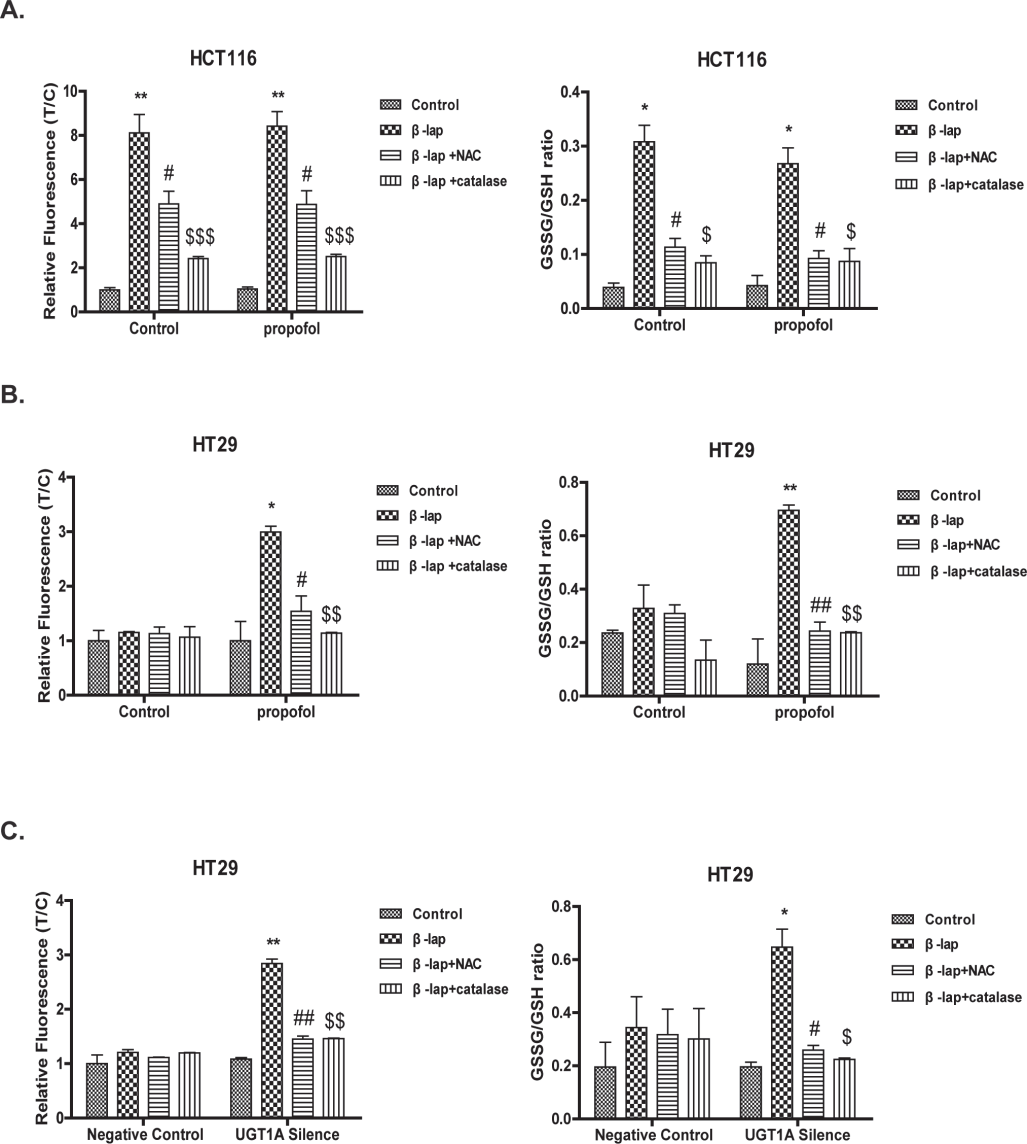
UGT1A Diminishes β-lap -induced ROS Formation. Cells were pretreated with UGT1A siRNA or scrambled siRNA (negative control) for 24 h, or pretreated with propofol (100 μM)/ NAC (5 μM)/ catalase (4000 U) for 1 h. Then, cells were exposed to β-lap (5 μM) for 2 h and ROS assay or GSSG/GSH ratio assay was performed. (A) ROS formation and GSSG/GSH ratio in HCT116 cells pretreated with propofol/NAC/catalase; (B) ROS formation and GSSG/GSH ratio in HT29 cells pretreated with propofol/NAC/catalase; (C) ROS formation and GSSG/GSH ratio in HT29 cells pretreated with siRNA/NAC/catalase. Results are presented as mean ± 3 SEM of at least three independent experiments (*P<0.05, **P<0.01, ***P<0.001, β-lap treatment vs. control cells; #P<0.05, ##P<0.01, NAC pretreatment vs. corresponding β-lap only; $P<0.05, $$P<0.01, $ $ $P<0.001, catalase pretreatment vs. corresponding β-lap only).

Based on our previous work, β-lap undergoes NQO1 mediated two-electron reduction, forming a highly unstable catechol intermediate which could experience glucuronidation with the presence of UGTs or reverted back to β-lap in the absence of UGTs [22]. In the latter process, abundance of ROS was produced which then initiates cell apoptosis [24]. To further confirm the relationship between β-lap’s glucuronidation and the subsequent consequences, we examined ROS formation by DCF staining assay in HT29 and HCT116 cells treated with β-lap. β-lap induced significant ROS formation in HCT116 cells, which could be largely combated by ROS eliminator N-Acetyl-L-cysteine (NAC) or catalase but not by UGT1A9 inhibitor propofol (Fig. 3A). However, β-lap induced ROS formation in HT29 cells was only observed when treated with propofol, which was also reduced by NAC or catalase (Fig. 3B). In addition, UGT1A siRNA transfection also significantly enhanced ROS levels in HT29 cells (Fig. 3C).

Reduced glutathione (L-γ-glutamyl-L-cysteinyl-glycine, GSH) is the biological active form that is oxidized to glutathione disulfide (GSSG) during oxidative stress, and the ratio of GSSG-to-GSH thus offers a simple and convenient expression of cellular oxidative stress [25,26]. We then evaluated cellular redox balance by testing GSSG/GSH in HT29 and HCT116 cells and found the same change tendency as DCF staining assays (Fig. 3A, Fig. 3B and Fig. 3C).

### UGT1A Affects β-lap induced Cytotoxicity in Colon Cancer Cells

**Figure 4:**
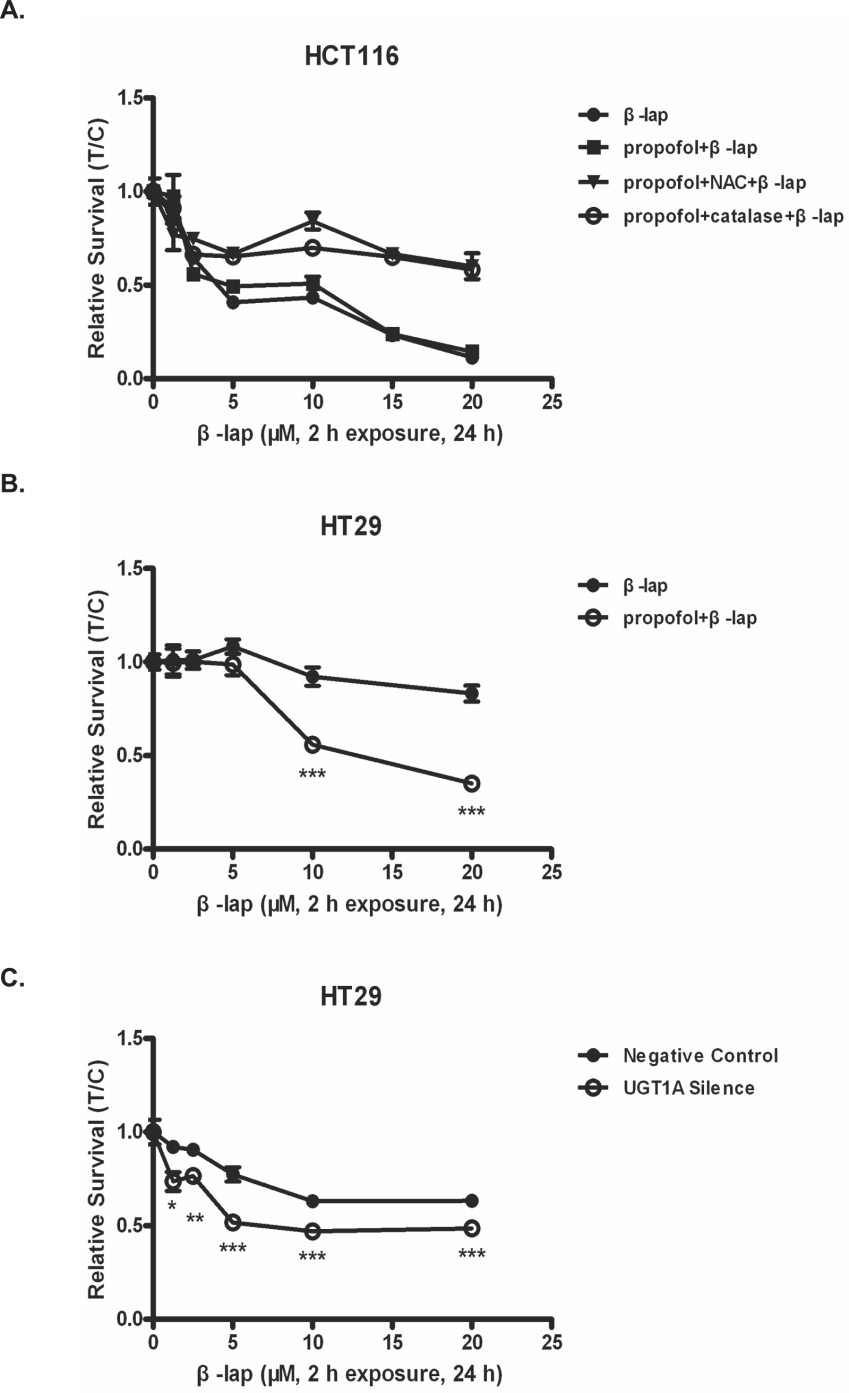
UGT1A compromises β-lap induced cytotoxicity. Cells were pretreated with propofol (100 μM) for 1 h or UGT1A siRNA for 24 h. Then, Cells were exposed to gradient concentrations of β-lap (0, 1.25, 2.5, 5, 10, 20 μM) for 24 h and MTT assay was performed. (A) β-lap induced cytotoxicity in HCT116 cells with propofol/NAC/catalase pretreatment; (B) β-lap induced cytotoxicity in HT29 cells with propofol/NAC/catalase pretreatment; (C) β-lap induced cytotoxicity in HT29 cells with siRNA/NAC/catalase pretreatment. Results are presented as mean ± 6 SEM of at least three independent experiments (*P<0.05, **P<0.01, ***P<0.001, propofol/siRNA pretreatment vs. β-lap only; #P<0.05, ##P<0.01, ###P<0.001, NAC and propofol/siRNA pretreatment vs. propofol/siRNA only; $ $P<0.01, $ $ $P<0.001, catalase and propofol/siRNA pretreatment vs. propofol/siRNA only).

Excessive ROS induces oxidative modification of cellular macromolecules, inhibits protein function and promotes cell death [27]. Since UGT1A was capable of inhibiting ROS formation, we then explored whether UGT1A activity influences β-lap induced cytotoxicity. Results showed that β-lap exhibited obvious cytotoxicity in HCT116 cells which was reversed by NAC and catalase but not by propofol (Fig. 4A). In contrast, β-lap cytotoxicity to HT29 cells was week but could be strengthened by pretreatment with propofol (Fig. 4B); NAC and catalase were capable of reversing this effect. Consistently, UGT1A siRNA transfections also enhanced the cytotoxic effect of β-lap in HT29 cells, which was significantly reversed by NAC and catalase (Fig. 4C).

### UGT1A Activity Causes Resistance of β-lap induced apoptosis in Colon Cancer Cells

**Figure 5:**
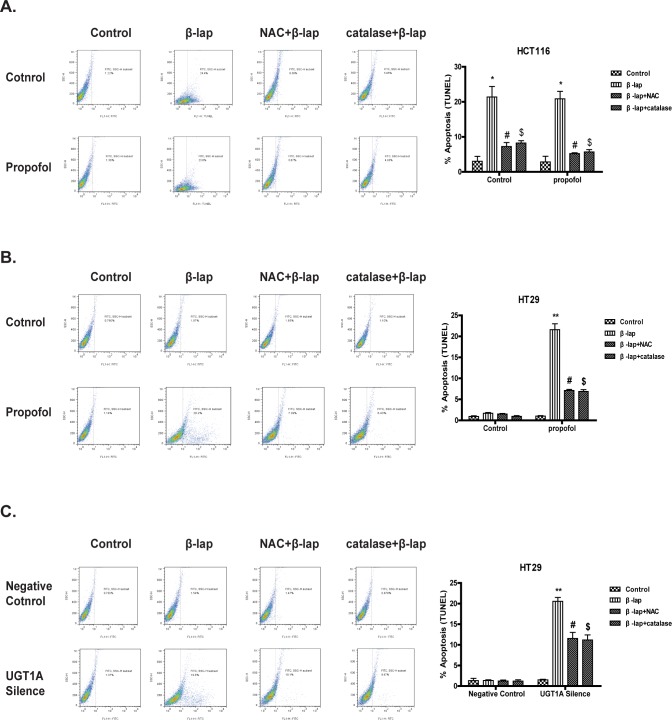
UGT1A activity affects β-lap -induced apoptosis to Colon Cancer Cells. Cells were pretreated with propofol (100 μM) for 1 h or UGT1A siRNA for 24 h. Then cells were exposed to β-lap (5 μM) for 24 h and collected. Cell apoptosis was assessed by TUNEL assay. (A) Cell apoptosis in HCT116 cells with propofol/NAC/catalase pretreatment; (B) Cell apoptosis in HT29 cells with propofol/NAC/catalase pretreatment; (C) Cell apoptosis in HT29 cells with siRNA/NAC/catalase pretreatment. Results are presented as mean ± 3 SEM of at least three independent experiments (*P<0.05, **P<0.01, β-lap treatment vs. control cells; #P<0.05, NAC pretreatment vs. corresponding β-lap only; $P<0.05, catalase pretreatment vs. corresponding β-lap only).

Cell apoptotic death was evaluated by TUNEL staining assay to further investigate the role of UGTs in β-lap’s anti-cancer efficacy. Colon cancer cells were pretreated with propofol or UGT1A siRNA to inhibit UGT1A9 activity and then exposed to 5 μM of β-lap for 24 h. Data indicated that β-lap induced a significant apoptosis in HCT116 cells, which could be reversed by NAC or catalase but not by propofol pretreatment (Fig. 5A). However, in HT29 cells, β-lap induced apoptotic death was barely observed after 24 h exposure. HT29 cells pretreated with propofol or UGT1A siRNA and then to β-lap showed a significant apoptotic cell death (Fig. 5B, Fig. 5C). Combination pretreatment of NAC or catalase with propofol or UGT1A siRNA largely canceled the apoptotic effect of β-lap together with propofol pretreatment or UGT1A siRNA transfection of HT29 cells (Fig. 5B, Fig. 5C), suggesting a dominant role of UGTs in β-lap induced apoptotic death.

### UGT1A represses β-lap -activated FOXO1 apoptotic pathway in colon cancer cells

**Figure 6:**
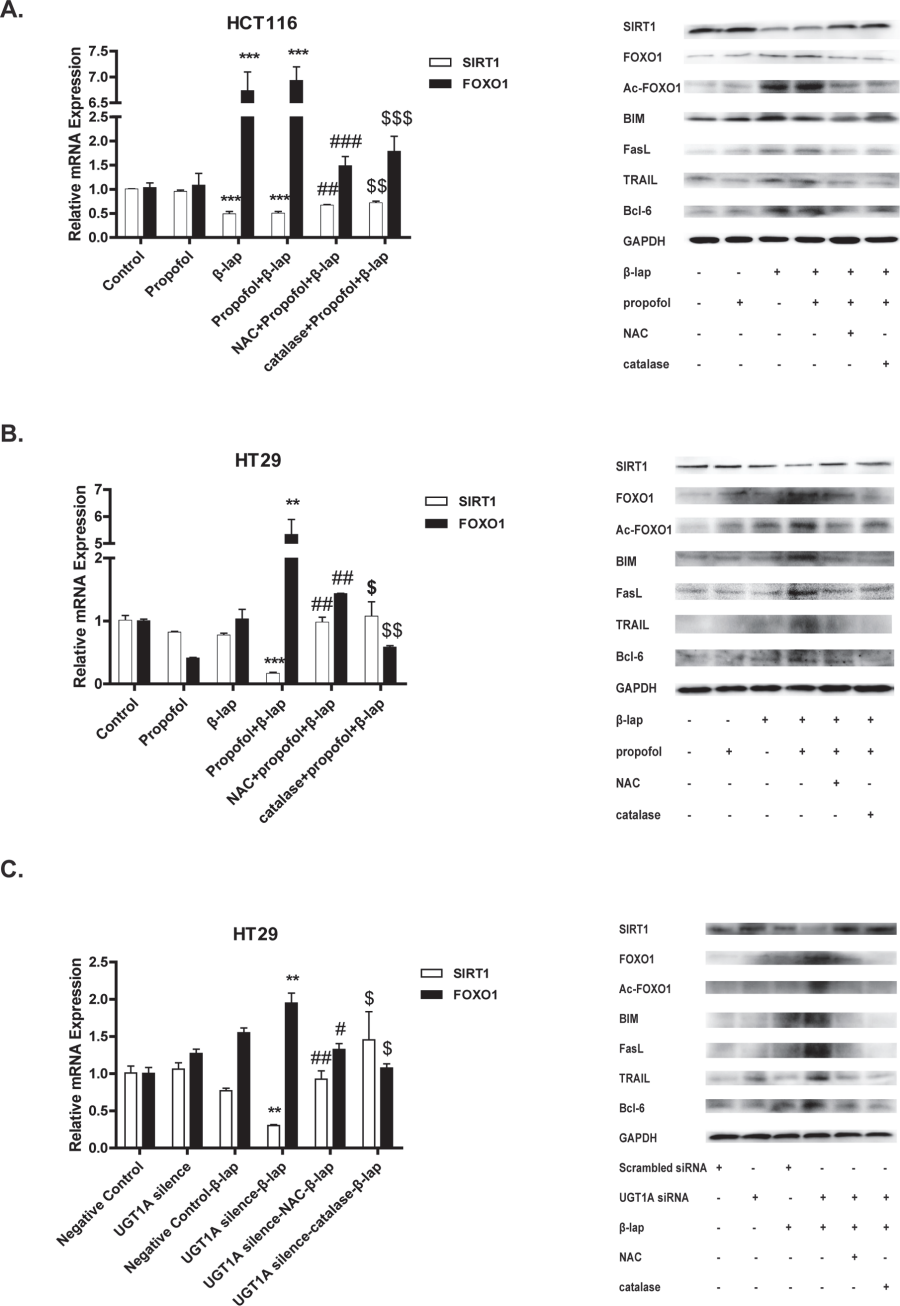
UGT1A compromises β-lap-activated FOXO1 apoptosis pathway in colon cancer cells. Cells were pretreated with propofol (100 μM) for 1 h or UGT1A siRNA for 24 h. Then, cells were exposed to β-lap (5 μM) for 24 h and collected. Cell mRNA levels were assessed by PCR and protein levels by western blot assays. (A) Relative mRNA and protein levels in HCT116 cells with propofol/NAC/catalase pretreatment; (B) Relative mRNA and protein levels in HT29 cells with propofol/NAC/catalase pretreatment; (C) Relative mRNA and protein levels in HT29 cells with siRNA/NAC/catalase pretreatment. Results are presented as mean ± 3 SEM of at least three independent experiments (*P<0.05, **P<0.01, ***P<0.001, propofol pretreatment vs. control cells, or UGT1A siRNA pretreatment vs. scrambled siRNA pretreatment; #P<0.05, ###P<0.001, NAC pretreatment vs. corresponding β-lap only; $P<0.05, $ $P<0.01, catalase pretreatment vs. corresponding β-lap only).

Our previous studies demonstrated that SIRT1-FOXO1 apoptotic pathway is involved in β-lap induced apoptotic cell death. β-lap causes NAD^+^ depletion, reduces the activity of NAD^+^-deacetylase silent mating type information regulation 2 homolog 1 (SIRT1), leading to reduced deacetylation of Forkhead box O 1 (FOXO1) and increased accumulation of acetylated-FOXO1 (Ac-FOXO1) and thereby the enhanced transcriptional activity towards multiple downstream apoptotic targets. To validate whether this pathway is also involved in UGT1A-repressed apoptotic effect of β-lap, we investigated the activation of SIRT1-FOXO1 pathway after β-lap exposure with or without propofol or UGT1A siRNA pretreatment. According to real-time PCR and western blot assays results, the expression of SIRT1 was greatly inhibited while FOXO1, Ac-FOXO1 as well as its downstream apoptotic target proteins (BIM, FasL, TRAIL, and Bcl-6) were all up-regulated after β-lap exposure in HCT116 cells (Fig. 6A); this effect could be largely revesed by NAC or catalase pretreatment but not by propofol (Fig. 6A). In contrast, in HT29 cells, β-lap alone could not reduce SIRT1 expression and evoke FOXO1 due to the rapid elimination of β-lap. However, similar results to those observed in HCT116 cells were found in HT29 cells pretreated with propofol or UGT1A siRNA, which was also counteracted by NAC or catalase (Fig. 6B, Fig. 6C).

## Discussion

β-lapachone (β-lap) is a promising anticancer agent by a mechanism of action highly dependent on the enzyme NQO1, a flavoprotein found overexpressed in various cancer cells [10,28,29]. Previous studies in our lab demonstrated that UGT1A9 and UGT2B7 are the main two UGT isoforms that catalyze the metabolism of β-lap in human liver and intestinal S9 incubations [22]. Though several reports have provided evidence for a glucosylsulfate conjugate metabolite of β-lap, glucuronidation is its dominant metabolism pattern. Since UGTs mediated glucuronidation accounts for multiple drug resistance in cancer therapy [30–32], we evaluated the potential role of UGTs mediated cellular metabolism of β-lap in determining its anti-cancer effects in human colon cancer cells. A pair of cell lines were chosen in our study, one with high levels of UGT1A (HT29) and another with no UGT1A expression (HCT116), which were also involved explorations on UGTs [23,33,34]. Glucuronidation metabolite M2, but not M1, of β-lap was detected in HT29 cells and S9 fractions, similar to that observed from the human intestinal S9 study [22]. This may be rationally explained by the low expression of UGT2B7 in human gastrointestinal tract [7]. UGT1A9 typical inhibitor propofol or UGT1A siRNA showed potent inhibitory effect on M2 formation, indicating UGT1A9 is the main isoform of β-lap glucuronidation in HT29 cells. Compared with HT29 cells, the lack of UGT1A9 in HCT116 cells leads to a significant higher AUC value of β-lap and undetectable M2. Either propofol or UGT1A siRNA pretreatment remarkably intensified intracellular accumulation of β-lap, supported by significant higher C_max_ and AUC values. It is worth mentioning that intracellular M2 levels dropped during the time course while that in culture medium increased continuously, indicating an elimination of β-lap metabolite from the cells to the extracellular medium.

As described in detail in our previous work, β-lap metabolism involves two steps [22]. The first step is two-electron reduction to produce a catechol intermediate, promoted by the two-electron quinone reduction enzyme NQO1. This process can be reversed automatically in a nonenzymatic mode, producing large amount of ROS [22,24]. In UGTs-positive cells, the catechol intermediate is subjected to immediate glucuronidation, thus breaking the redox cycle by diverting to the production of stable β-lap glucuronides [22]. Therefore, UGT1A not only determines intracellular accumulation of β-lap, but more importantly can disrupt NQO1-triggered redox cycle that is the dominant mechanism for β-lap to elicit its anti-tumor efficacy. This fact suggests that the expression of UGT1A in cancer cells may represent an important mechanism underlying the intrinsic resistance of β-lap. To test this hypothesis, we examined the potential influence of UGT1A in β-lap-induced ROS production, cytotoxicity, apoptosis and apoptotic signal transduction. As presumed, β-lap triggered significant ROS production, cellular redox homeostasis disorder, and strong cytotoxicity at very low concentration and short time exposure in HCT116 cells. In contrast, ROS production and the resultant cytotoxicity in HT29 cells was only observed in the presence of propofol or UGT1A siRNA, providing strong evidence to that UGT1A is an important determinant in the cytotoxic effect of β-lap.

Next, we extended the study to determine the potential influence of UGT1A in disrupting the apoptotic cell death pathway activated by β-lap. Multiple modes and mechanisms of β-lap induced cell death including apoptosis, programmed necrosis, and autophage have been reported [10,35,36]. Here, we found that β-lap mainly induced a typical apoptotic death of human colon cancer cell lines. Sirtuin, a conserved family of NAD^+^-dependent deacetylases and mono-ADP-ribosyltransferases, has been found involved in diverse cellular processes such as gene silencing, DNA repair, life span extension and cell apoptosis [37–39]. β-lap induced apoptotic cell death is characterized with a dramatic NAD^+^ depletion and reduced SIRT1 activity, leading to the increasing accumulation of acetylated-FOXO1 and thereafter the transcriptional activation of its downstream apoptotic target genes in HCT116 cells. In contrast, the activation of this typical apoptotic pathway in HT29 cells was only observed with propofol or UGT1A siRNA pretreatment. The impairment of β-lap’s ability to activate SIRT1-FOXO1 apoptotic pathway in HT29 cells due to glucuronidation hints that UGTs high expression in cancer cells may affect the responses to many therapeutic drugs. Moreover, both NAC and catalase treatment largely prevented the activation of this apoptotic cell death signal, suggesting that the ROS production from NQO1-triggered redox cycle may be the important casual factor for the apoptotic effect of β-lap and that UGT1A may cause intrinsic resistance via diverting the redox cycle.

In summary, we demonstrated that UGT1A plays a vital role in intracellular accumulation and the resultant apoptotic effect of β-lap in colon cancer cells. Lack of UGTs activity raises the possibility of continuous redox cycle of β-lap in which excessive ROS is produced and finally leads to apoptotic cell death. On the contrary, high levels of UGT1A activity, especially UGT1A9, may break this cycle and promote β-lap’s metabolic elimination. The present study together with our previous work hint to that the anti-cancer effects of NQO1 targeting agents are largely associated with the balance of expression and activity of NQO1 and UGT1A. Notably, tumor tissues usually bear much higher NQO1 [40,41] but lower UGTs [5,42] than in normal tissues. This property confers β-lap as well as other NQO1 targeting agents such as Tanshinone IIA [7,24] a feature of high selectivity in killing cancer cells while sparing normal tissues. Together with our previous study on Tanshinone IIA, the current work on β-lap further highlights that UGTs medicated metabolism may represent a pivotal mechanism in causing intrinsic chemoresistance of NQO1 targeting agents that are usually UGTs’ substrates. It is also important to note that the intrinsic and/or inductive expression of UGT1A in cancer cells may confer resistance to NQO1 targeting substrates.

## Supporting Information

S1 Fig UGT1A expression in colon cancer cells. (A) mRNA levels of UGT1A isoforms in HT29 cells treated with UGT1A siRNA or scrambled siRNA; (B) Protein levels of UGT1A in HT29 and HCT116 cells; (C) Protein levels of UGT1A in HT29 cells treated with UGT1A siRNA or scrambled siRNA. Results are presented as mean ± 3 SEM of at least three independent experiments.
(DOCX)
