# Supplementary material for: UDP-Glucuronosyltransferase 1A Determinates Intracellular Accumulation and Anti-Cancer Effect of β-Lapachone in Human Colon Cancer Cells
Source: PLoS One. 2015 Feb 18;10(2):e0117051. doi: 10.1371/journal.pone.0117051 (PMC4333567; doi:10.1371/journal.pone.0117051)
Supplement: S1 Fig — (A) mRNA levels of UGT1A isoforms in HT29 cells treated with UGT1A siRNA or scrambled siRNA; (B) Protein levels of UGT1A in HT29 and HCT116 cells; (C) Protein levels of UGT1A in HT29 cells treated with UGT1A siRNA or scrambled siRNA. Results are presented as mean ± 3 SEM of at least three independent experiments. (DOCX) [file pone.0117051.s001.docx]

###
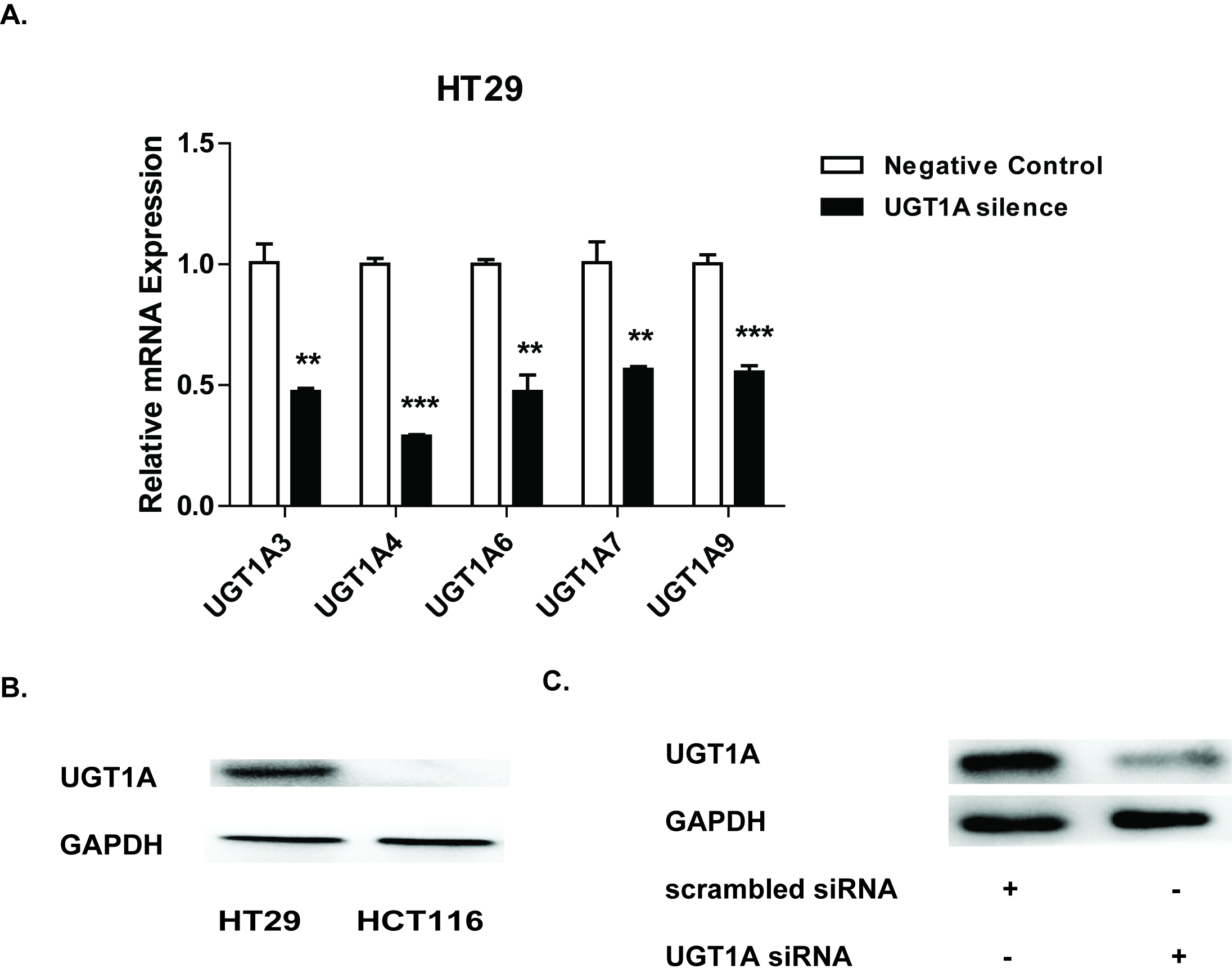
 Supplementary Fig.1 UGT1A expression in colon cancer cells. (A) mRNA levels of UGT1A isoforms in HT29 cells treated with UGT1A siRNA or scrambled siRNA; (B) Protein levels of UGT1A in HT29 and HCT116 cells; (C) Protein levels of UGT1A in HT29 cells treated with UGT1A siRNA or scrambled siRNA. Results are presented as mean ± 3 SEM of at least three independent experiments.
